# Efficacy and safety of re-irradiation for locoregional esophageal squamous cell carcinoma recurrence after radiotherapy: a systematic review and meta-analysis

**DOI:** 10.1186/s13014-022-02019-0

**Published:** 2022-03-28

**Authors:** Kuntian Lan, Jiaohong Chen

**Affiliations:** Department of Radiotherapy, Cancer Branch of Sanmenxia Central Hospital, Sanmenxia, 472000 China

**Keywords:** Re-irradiation, Esophageal squamous cell carcinoma, Recurrence, Radiotherapy, Systematic review, Meta-analysis

## Abstract

**Background:**

There is currently no standard treatment for locoregional recurrence of esophageal squamous cell carcinoma (ESCC) previously treated with radiotherapy. This study aimed to assess the efficacy and safety of re-irradiation for ESCC patients with locoregional recurrence.

**Methods:**

The PubMed, EmBase, and Cochrane library databases were systematically searched for eligible studies published before January 2021. The pooled effect estimates were calculated using the random effects model. Subgroup analyses were conducted to assess the treatment effectiveness of re-irradiation based on specific characteristics.

**Results:**

Nine retrospective studies including 573 ESCC patients with locoregional recurrence were selected. The pooled incidences of the 1-year, 2-year, 3-year, and 5-year survival for patients after re-irradiation were 59% (95% confidence interval [CI]: 35–83; *P* < 0.001), 25% (95% CI: 16–33; *P* < 0.001), 25% (95% CI: 4–45; *P* = 0.017), and 15% (95% CI: 2–27; *P* = 0.024), respectively. The rates of complete response and local re-recurrence after re-irradiation were 54% (95% CI: 21–88; *P* = 0.001) and 62% (95% CI: 55–70; *P* < 0.001), respectively. The median overall survival and local failure-free survival for patients after re-irradiation were 13.94 months (95% CI: 4.18–46.51; *P* < 0.001) and 11.01 months (95% CI: 5.99–20.22; *P* < 0.001), respectively. Grade ≥ 3 adverse events of esophageal perforation, tracheoesophageal fistula, and radiation pneumonitis were significantly more common after re-irradiation.

**Conclusions:**

This study found that re-irradiation for ESCC patients with locoregional recurrence after previous radiotherapy was feasible. However, patients should be carefully observed in order to treat associated adverse events, including esophageal perforation, tracheoesophageal fistula, and radiation pneumonitis.

**Supplementary Information:**

The online version contains supplementary material available at 10.1186/s13014-022-02019-0.

## Introduction

Esophageal cancer is the eighth most common cancer worldwide, with more than 450,000 new cases annually, and the sixth leading cause of cancer-related deaths [1]. Esophageal squamous cell carcinoma (ESCC) is the predominant histological type of esophageal cancer [2]. Although patients with early ESCC undergo esophagectomy, most ESCC patients are diagnosed with locally advanced disease. Therefore, surgery alone is usually insufficient, and radiotherapy or chemotherapy are administered [3, 4]. However, the prognosis of ESCC remains poor, and local recurrence or metastasis play a critical role in treatment failure and mortality [5].

Previous studies have shown that local recurrence is the major cause of ESCC recurrence after definitive chemoradiotherapy, which accounts for nearly 40–60% of ESCC cases [6, 7]. Re-irradiation is a common salvage treatment strategy and is widely administered for recurrent ESCC after radical radiotherapy or chemoradiotherapy. Chen et al. found that re-irradiation was able to relieve symptoms and prolong survival [8]. However, the efficacy of re-irradiation for locoregional recurrent ESCC previously treated with radiotherapy is variable, and whether the efficacy of re-irradiation is affected by patient characteristics remains controversial. We therefore performed a systematic review and meta-analysis to assess the efficacy and safety of re-irradiation for ESCC patients with locoregional recurrence who were previously treated with radiotherapy.

## Methods

### Search strategy and selection criteria

The Preferred Reporting Items for Systematic Reviews and Meta-Analysis guideline was used to guide this systematic review and meta-analysis [9]. Studies investigating the treatment effects of re-irradiation for patients with locoregional recurrent ESCC previously treated with radiotherapy were eligible. Only studies published in English were included. The PubMed, EmBase, and Cochrane library databases were searched for eligible studies published through January 2021, and the following search terms were used: esophageal squamous cell carcinoma, recurrence or recurrent, and radiotherapy or irradiation. We also manually reviewed the reference lists of retrieved studies to identify any additional studies that met the inclusion criteria.

The inclusion criteria were as follows: (1) Study design: retrospective, prospective, or randomized controlled trials; (2) Patients: patients with recurrent ESCC previously treated with definitive radiotherapy; (3) Intervention: re-irradiation; and (4) Outcomes: survival rate at 1, 2, 3, or 5 years, complete response, local re-recurrence, overall survival, local failure-free survival, and grade ≥ 3 adverse events. Study selection was performed by two reviewers, and conflicts between reviewers was settled by group discussion until a consensus was reached.

#### Data collection and quality assessment

Two reviewers independently extracted the following information: first author’s name, publication year, country, study design, sample size, mean age, male proportion, tumor stage, interval from prior therapy to irradiation, total radiation dose, and reported outcomes. The quality of each individual study was assessed using the Newcastle–Ottawa Scale, and the starring system for each study ranged from 0 to 9 [10]. Studies with 6 stars were considered to be of moderate quality, and studies with 4 or 5 stars were regarded as low quality. Inconsistent results between reviewers for extracted data and quality assessment were settled by an additional reviewer after referring to the original article.

#### Statistical analysis

The pooled incidence of survival at 1, 2, 3, and 5 years, complete response, local re-recurrence, and grade ≥ 3 adverse events were calculated based on the number of events and total patients, whereas pooled overall survival and local failure-free survival were calculated based on mean survival and 95% confidence intervals (CIs). The pooled effect estimates were calculated using the random effects model, and underlying variations across included studies were considered [11, 12]. Heterogeneity among the included studies for each outcome was assessed using I2 and the Q statistic, and significant heterogeneity was defined as I^2^ > 50.0% or *P* < 0.10 [13, 14]. The robustness of the pooled conclusion was assessed using sensitivity analysis by sequentially removing a single study [15]. Subgroup analyses for survival at 1, 2, 3, and 5 years were also performed according to sample size, mean age, male proportion, tumor stage, interval from prior therapy to radiotherapy, and study quality, and the differences between subgroups were assessed using the interaction *P* test [16]. Funnel plot, Egger test, and Begg’s tests were used to assess potential publication bias [17, 18]. The *P* values for pooled conclusions are two-sided, and the inspection level was 0.05. All statistical analyses in this study were performed using STATA software (version 10.0; Stata Corporation, College Station, TX, USA).

## Results

### Literature search

**Fig. 1:**
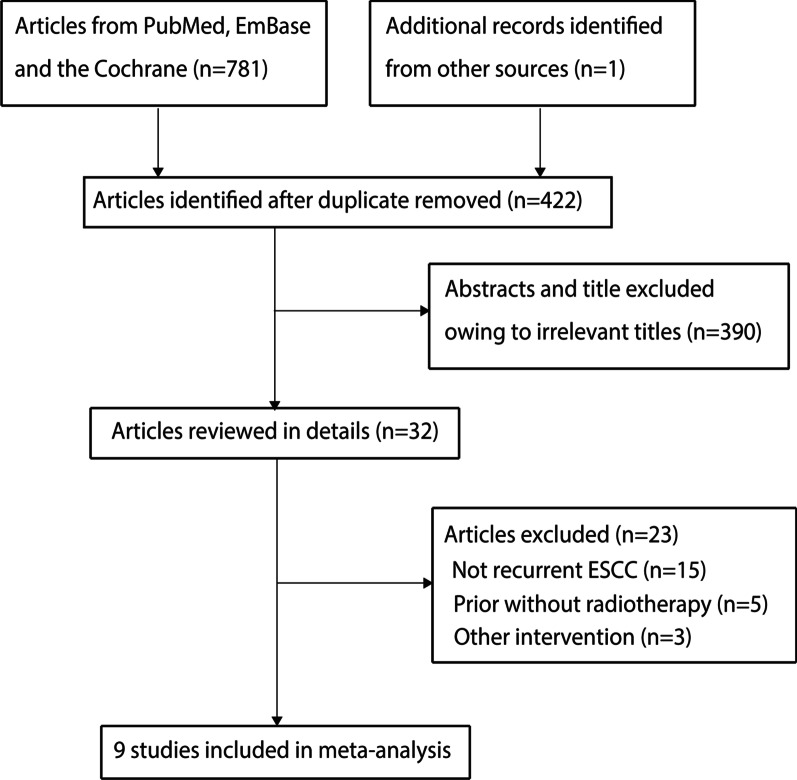
The PRISMA flowchart for study selection process

A total of 781 articles were identified from initial searches of the PubMed, EmBase, and Cochrane library databases, and 422 studies were retained after duplicate titles were removed. A further 390 studies were excluded because they covered irrelevant topics. The remaining 32 studies were retrieved for full-text evaluation, and 23 studies were excluded for the following reasons: not recurrent ESCC (n = 15), patients were previously without radiotherapy (n = 5), and other interventions (n = 3). Reviewing the reference lists yielded one additional potential study, but this study was also retrieved by our electronic search. Finally, nine studies were selected for the final meta-analysis (Fig. 1) [19–27].

#### Study characteristics

The characteristics of the included studies and patients are summarized in Table 1. All of the studies were retrospective, and a total of 573 ESCC patients with locoregional recurrence were included. Four studies were conducted in Japan, four studies were conducted in China, and one study was conducted in Korea. The mean age for patients ranged from 60.8 years to 72.5 years, and the male proportion ranged from 49.1 to 100.0%. Four studies were of moderate quality, and the remaining five studies were of low quality.

**Table 1:** The baseline characteristics of the eligible studies and included patients

Study	Country	Study design	Sample size	Age (years)	Male (%)	Stage (I–II/III–IV)	Interval from prior therapy to irradiation	Total radiation dose	Study quality
Shioyama 2007 [19]	Japan	Retrospective	82	61.0	85.4	57/24	10.0 months	50.4 Gy	6
Nonoshita 2007 [20]	Japan	Retrospective	6	70.5	100.0	6/0	4.3 months	22.8 Gy	4
Maruyama 2011 [21]	Japan	Retrospective	28	64.2	92.9	12/16	NA	60 Gy	4
Kim 2012 [22]	Korea	Retrospective	10	72.5	90.0	6/4	15.6 months	46.5 Gy	5
Zhou 2015 [23]	China	Retrospective	55	66.8	49.1	54/1	NA	51.9 Gy	6
Katano 2017 [24]	Japan	Retrospective	6	60.8	100.0	NA	25.2 months	50.4 Gy	4
Hong 2018 [25]	China	Retrospective	87	62.0	74.7	23/47	16.0 months	60 Gy	5
Xu 2019 [26]	China	Retrospective	47	72.0	74.5	24/23	26.0 months	58 Gy	6
Zhao 2020 [27]	China	Retrospective	252	69.0	81.7	79/173	30.0 months	72 Gy	6

#### Survival rate at 1, 2, 3, and 5 years

**Fig. 2:**
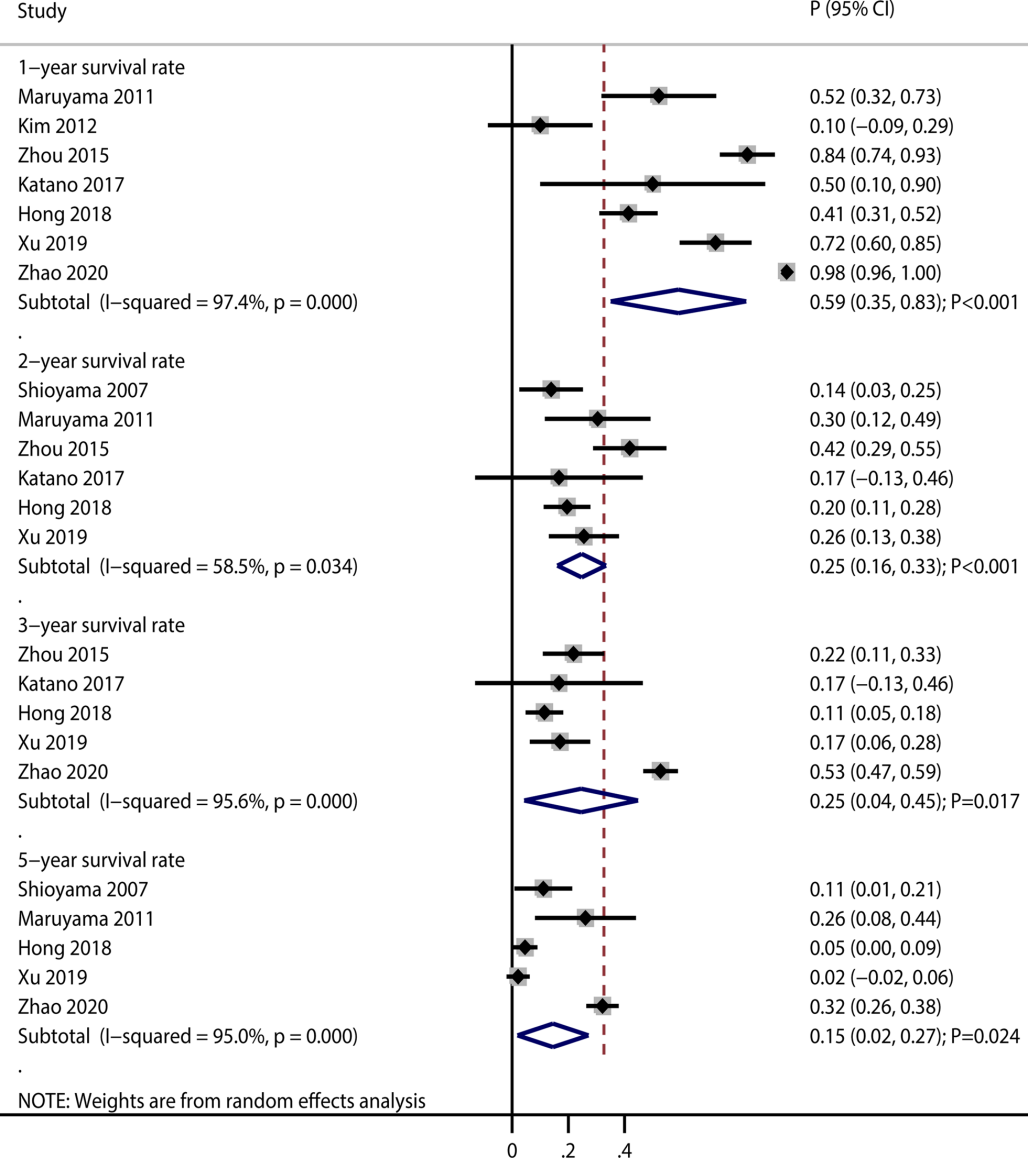
The pooled incidences of survival at 1, 2, 3, and 5 years after re-irradiation

Six, five, four, and five studies reported the effects of re-irradiation on survival at 1, 2, 3, and 5 years, respectively (Fig. 2). The pooled incidences of 1-year, 2-year, 3-year, and 5-year survival for patients after re-irradiation were 59% (95% confidence interval [CI]: 35–83; *P* < 0.001), 25% (95% CI: 16–33; *P* < 0.001), 25% (95% CI: 4–45; *P* = 0.017), and 15% (95% CI: 2–27; *P* = 0.024), respectively. There was significant heterogeneity for the survival rates at 1 year (I^2^ = 97.4%; *P* < 0.001), 2 years (I^2^ = 58.5%; *P* = 0.034), 3 years (I^2^ = 95.6%; *P* < 0.001), and 5 years (I^2^ = 95.0%; *P* < 0.001). Sensitivity analyses found the ranges for survival at 1, 2, 3, and 5 years were 52.3–67.7%, 20.2–27.4%, 15.0–28.2%, and 6.7–18.1%, respectively (Additional file 1). There was potential significant publication bias for the 1-year survival rate (Egger test *P*: 0.017; Begg’s test *P*: 0.548), but there was no significant publication bias for the 2-year (Egger test *P*: 0.625; Begg’s test *P*: 0.452), 3-year (Egger test *P*: 0.595; Begg’s test *P*: 0.806), or 5-year (Egger test *P*: 0.477; Begg’s test *P*: 0.221) survival rates (Additional file 1).

#### Complete response and local re-recurrence

**Fig. 3:**
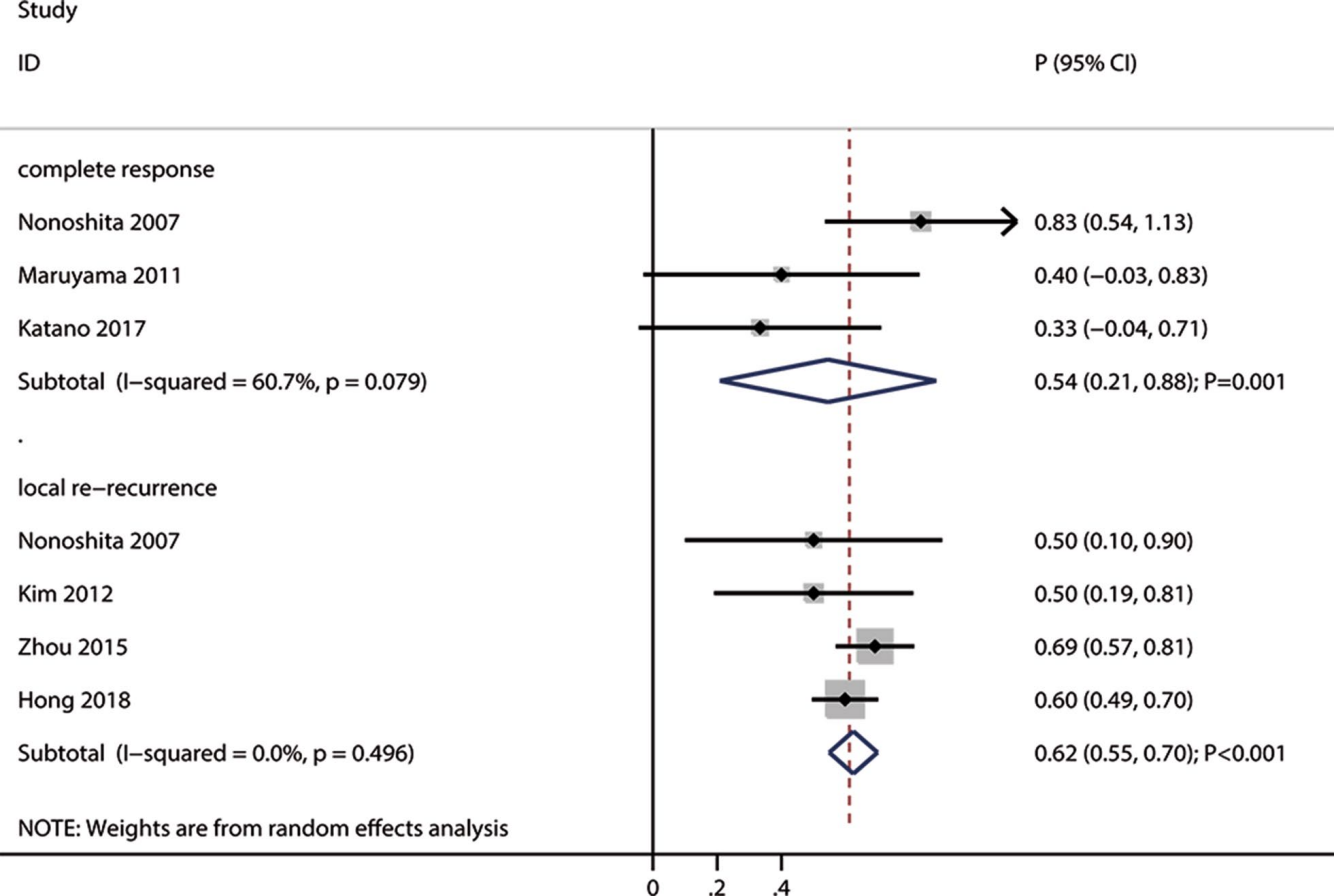
The pooled incidences of complete response and re-recurrence after re-irradiation

Three and four studies reported the effects of re-irradiation on complete response and local re-recurrence, respectively (Fig. 3). The pooled incidences of complete response and local re-recurrence after re-irradiation were 54% (95% CI: 21–88; *P* = 0.001) and 62% (95% CI: 55–70; *P* < 0.001), respectively. There was significant heterogeneity for complete response (I^2^ = 60.7%; *P* = 0.079), but no evidence of heterogeneity was observed for local re-recurrence (I^2^ = 0.0%; *P* = 0.496).

#### Overall survival and local failure-free survival

**Fig. 4:**
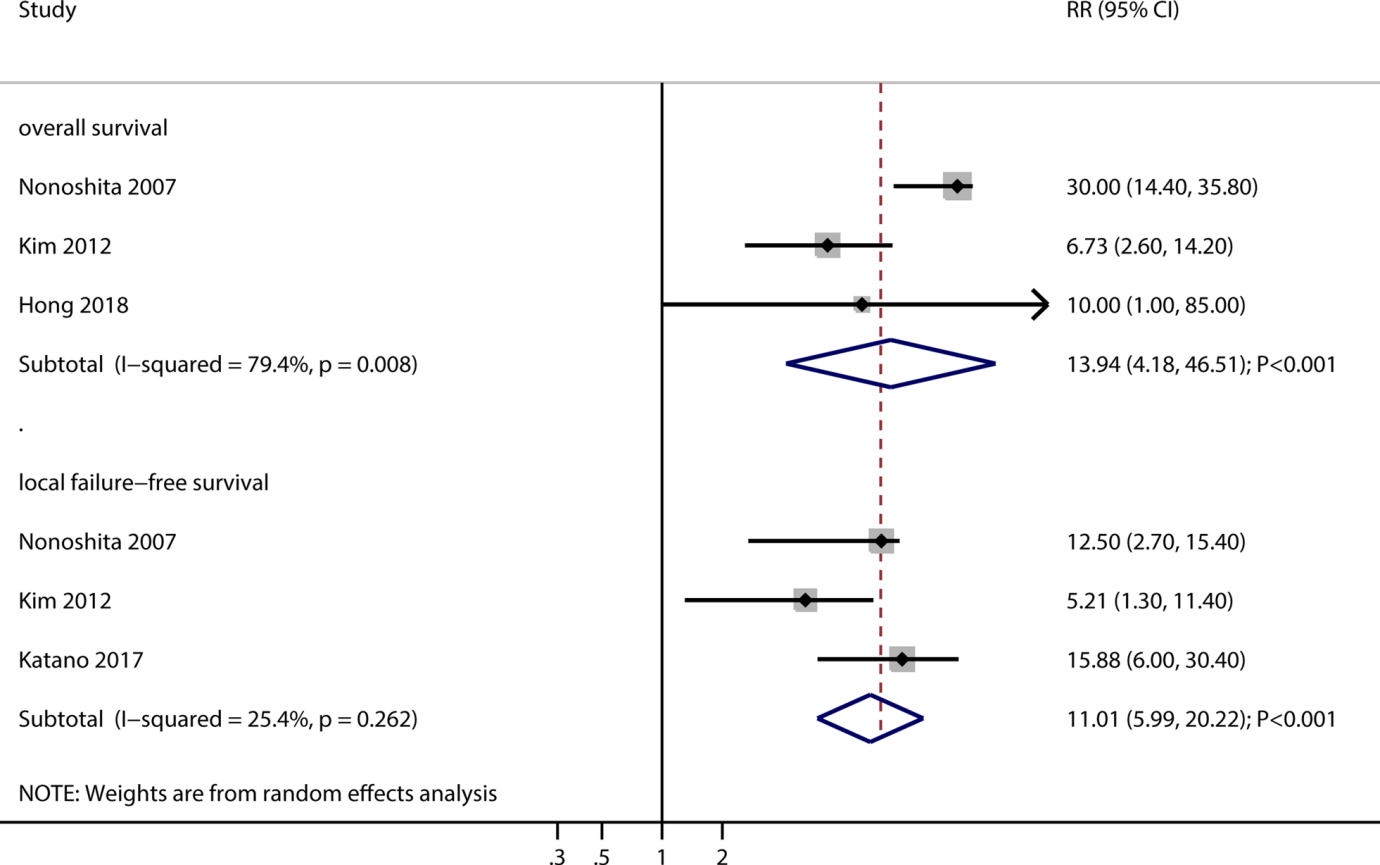
The pooled overall survival and local failure-free survival after re-irradiation

Three and three studies reported the effects of re-irradiation on overall survival and local failure-free survival, respectively (Fig. 4). The pooled overall survival and local failure-free survival after re-irradiation were 13.94 months (95% CI: 4.18–46.51; *P* < 0.001) and 11.01 months (95% CI: 5.99–20.22; *P* < 0.001), respectively. There was significant heterogeneity for overall survival (I^2^ = 79.4%; *P* = 0.008) but not for local failure-free survival (I^2^ = 25.4%; *P* = 0.262).

#### Grade ≥ 3 adverse events

**Fig. 5:**
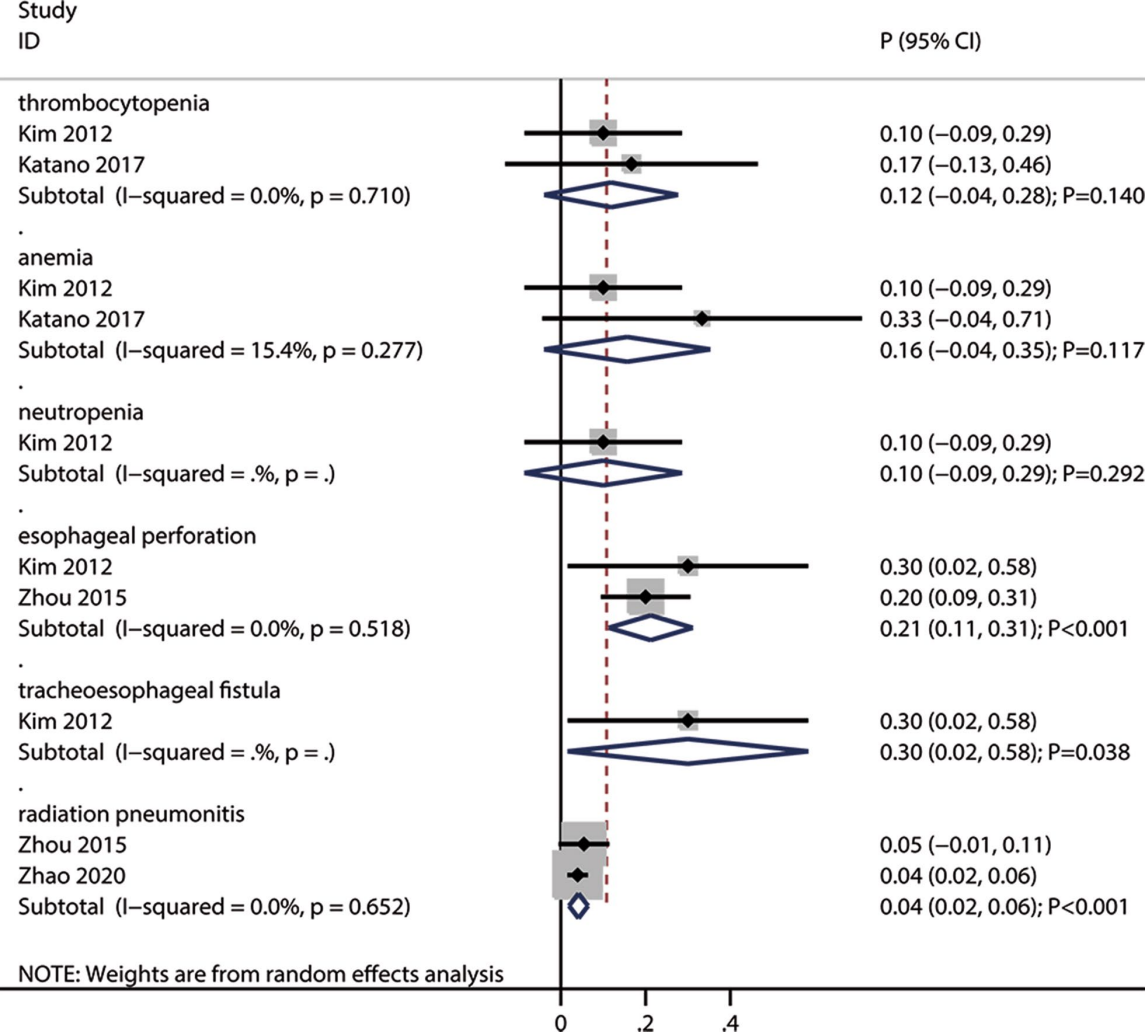
The pooled grade ≥ 3 adverse events after re-irradiation

Esophageal perforation (pooled incidence: 21%; 95% CI: 11–31; *P* < 0.001), tracheoesophageal fistula (pooled incidence: 30%; 95% CI: 2–58; *P* = 0.038), and radiation pneumonitis (pooled incidence: 4%; 95% CI: 2–6; *P* < 0.001) were significantly more common after re-irradiation (Fig. 5). However, re-irradiation was not associated with increased incidences of thrombocytopenia, anemia, or neutropenia. There was no significant heterogeneity for grade ≥ 3 adverse events.

#### Subgroup analysis

The results of subgroup analyses for survival at 1, 2, 3, and 5 years are shown in Table 2. The 1-year survival rate after re-irradiation was higher in studies with a sample size ≥ 50, mean age ≥ 65 years, male proportion < 90%, ≥ 50% patients with tumor stage III and IV, mean interval from prior therapy to radiotherapy ≥ 24 months, and moderate quality. The 2-year survival rate after re-irradiation was higher in studies with a mean age ≥ 65 years and mean interval from prior therapy to radiotherapy ≥ 24 months. The 3-year survival rate after re-irradiation was higher in studies with a sample size ≥ 50, mean age ≥ 65 years, ≥ 50% patients with tumor stage III and IV, mean interval from prior therapy to radiotherapy ≥ 24 months, and moderate quality. The 5-year survival rate after re-irradiation was higher in studies with a sample size ≥ 50, mean age ≥ 65 years, ≥ 50% patients with tumor stage III and IV, mean interval from prior therapy to radiotherapy ≥ 24 months, and moderate quality.

**Table 2:** Subgroup analyses for survival at 1, 2, 3, and 5 years

Outcomes	Factors	Groups	Number of studies	Incidence and 95% CI	*P* value	I^2^ (%)	*P* value for Q statistic	*P* value between subgroups
1-year survival rate	Sample size	≥ 50	3	0.74 (0.42–1.00)	< 0.001	98.3	< 0.001	< 0.001
		< 50	4	0.46 (0.15–0.78)	0.004	89.8	< 0.001	
	Mean age (years)	≥ 65	4	0.67 (0.41–0.94)	< 0.001	97.1	< 0.001	< 0.001
		< 65	3	0.44 (0.35–0.53)	< 0.001	0.0	0.622	
	Male (%)	≥ 90	3	0.36 (0.04–0.67)	0.026	79.8	0.007	< 0.001
		< 90	4	0.74 (0.48–1.00)	< 0.001	97.7	< 0.001	
	Stage III–IV (%)	≥ 50	3	0.64 (0.21–1.00)	0.004	98.4	< 0.001	< 0.001
		< 50	3	0.56 (0.19–0.93)	0.003	95.8	< 0.001	
	Interval from prior therapy to RT	≥ 24	3	0.79 (0.55–1.00)	< 0.001	90.0	< 0.001	< 0.001
		< 24	2	0.27 (− 0.04 to 0.57)	0.088	88.0	0.004	
	Study quality	Moderate	3	0.86 (0.70–1.00)	< 0.001	90.8	< 0.001	< 0.001
		Low	4	0.37 (0.18–0.56)	< 0.001	73.5	0.010	
2-year survival rate	Sample size	≥ 50	3	0.25 (0.10–0.39)	0.001	82.1	0.004	0.576
		< 50	3	0.26 (0.16–0.36)	< 0.001	0.0	0.743	
	Mean age (years)	≥ 65	2	0.34 (0.18–0.50)	< 0.001	68.1	0.077	0.010
		< 65	4	0.19 (0.13–0.25)	< 0.001	0.0	0.523	
	Male (%)	≥ 90	2	0.27 (0.11–0.42)	0.001	0.0	0.444	0.696
		< 90	4	0.25 (0.14–0.35)	< 0.001	73.5	0.010	
	Stage III–IV (%)	≥ 50	2	0.22 (0.13–0.30)	< 0.001	7.2	0.299	0.635
		< 50	3	0.27 (0.11–0.43)	0.001	80.1	0.006	
	Interval from prior therapy to RT	≥ 24	2	0.24 (0.13–0.36)	< 0.001	0.0	0.591	0.006
		< 24	2	0.18 (0.11–0.24)	< 0.001	0.0	0.430	
	Study quality	Moderate	3	0.27 (0.11–0.43)	0.001	80.1	0.006	0.365
		Low	3	0.21 (0.14–0.28)	< 0.001	0.0	0.558	
3-year survival rate	Sample size	≥ 50	3	0.29 (0.01–0.57)	0.045	97.6	< 0.001	0.007
		< 50	2	0.17 (0.07–0.27)	0.001	0.0	0.983	
	Mean age (years)	≥ 65	3	0.31 (0.06–0.56)	0.015	95.5	< 0.001	< 0.001
		< 65	2	0.12 (0.05–0.18)	< 0.001	0.0	0.740	
	Male (%)	≥ 90	1	0.17 (− 0.13 to 0.46)	0.273	–	–	0.381
		< 90	4	0.26 (0.04–0.48)	0.023	96.6	< 0.001	
	Stage III–IV (%)	≥ 50	2	0.32 (− 0.08 to 0.73)	0.119	98.7	< 0.001	0.004
		< 50	2	0.19 (0.12–0.27)	< 0.001	0.0	0.539	
	Interval from prior therapy to RT	≥ 24	3	0.30 (0.01–0.59)	0.042	94.3	< 0.001	< 0.001
		< 24	2	0.16 (0.06–0.26)	0.002	59.9	0.114	
	Study quality	Moderate	3	0.31 (0.06–0.56)	0.015	95.5	< 0.001	< 0.001
		Low	2	0.12 (0.05–0.18)	< 0.001	0.0	0.740	
5-year survival rate	Sample size	≥ 50	3	0.16 (− 0.03 to 0.35)	0.102	96.4	< 0.001	< 0.001
		< 50	2	0.12 (− 0.11 to 0.36)	0.294	84.6	0.011	
	Mean age (years)	≥ 65	2	0.17 (− 0.12 to 0.46)	0.255	98.5	< 0.001	0.031
		< 65	3	0.11 (0.01–0.21)	0.032	67.0	0.048	
	Male (%)	≥ 90	1	0.26 (0.08–0.44)	0.004	–	–	0.074
		< 90	4	0.12 (− 0.01 to 0.26)	0.077	96.1	< 0.001	
	Stage III–IV (%)	≥ 50	3	0.21 (− 0.01 to 0.42)	0.062	96.5	< 0.001	< 0.001
		< 50	2	0.05 (− 0.03 to 0.14)	0.215	60.5	0.112	
	Interval from prior therapy to RT	≥ 24	2	0.17 (− 0.12 to 0.46)	0.255	98.5	< 0.001	0.009
		< 24	2	0.06 (0.01–0.12)	0.027	23.4	0.253	
	Study quality	Moderate	3	0.15 (− 0.06 to 0.36)	0.156	97.1	< 0.001	0.019
		Low	2	0.14 (− 0.07 to 0.34)	0.202	80.8	0.023	

## Discussion

In this meta-analysis, we included nine studies assessing the effectiveness of re-irradiation in 573 ESCC patients with locoregional recurrence, and the effect estimates varied across the included studies. Although patients should be monitored for grade ≥ 3 esophageal perforation, tracheoesophageal fistula, and radiation pneumonitis in clinical practice, re-irradiation was effective for locoregional recurrent ESCC previously treated with radiotherapy. Moreover, the treatment effects of re-irradiation on survival rate were affected by sample size, mean age, male proportion, tumor stage, interval from prior therapy to radiotherapy, and study quality.

To our knowledge, this is the first meta-analysis focused on the treatment effects of re-irradiation for locoregional recurrent ESCC previously treated with radiotherapy. Several features of this study should be mentioned. First, it mainly included studies with lower quality, which restricted the representativeness of the cohort, and lower comparability, which could affect the reliability of the pooled results. Second, the heterogeneity in the survival rate at various timepoints was substantial, which could be explained by differences in tumor stage at initial diagnosis, initial treatment strategy, and radiation dose. Therefore, the results of this study should be generalized cautiously, and potential prognostic factors should be explored in further studies.

Our results on the pooled incidences of survival and recurrence after re-irradiation showed that re-irradiation is feasible. Although most studies suggested that re-irradiation could improve the survival rate at various timepoints, several studies reported a lower survival rate than expected [22, 24]. Kim et al. found an overall survival longer than 12 months in only one patient, and the mean overall survival was 6.73 months [22]. However, three patients experienced grade 5 tracheoesophageal fistula. Moreover, in the study by Katano et al., only one patient survived longer than 2 years [24]. Furthermore, two included studies found that the incidence of complete response, which was lower than expected and the pooled conclusion was variable [21, 24].

With regard to the safety of re-irradiation, our results showed that grade ≥ 3 esophageal perforation, tracheoesophageal fistula, and radiation pneumonitis were more common after re-irradiation. However, whether these adverse events were related to re-irradiation is unclear. The dose of irradiation for ESCC patients with locoregional recurrence after initial radiotherapy is important and needs further investigation [28].

Subgroup analyses showed that the survival rate after re-irradiation was affected by sample size, mean age, male proportion, tumor stage, interval from prior therapy to radiotherapy, and study quality. Interestingly, re-irradiation provided better survival outcomes for patients with high risk. There could be several reasons for these results. (1) The sample size was related to the power and weight from the overall analysis, which affected the robustness of the pooled conclusion. (2) Patient age is significantly related to disease severity and treatment tolerability. (3) The effect of radiosensitivity for ESCC patients might differ in men and women. Moreover, androgen could facilitate the growth of human ESCC cells, and the activation of androgen receptors could induce the progression of ESCC [29–31]. Therefore, the association of androgen levels with the prognosis of ESCC after radiotherapy needs to be further explored. (4) The baseline tumor stage before chemoradiotherapy or radiotherapy is a significant prognostic factor for ESCC [32]. (5) The interval from prior therapy to radiotherapy is significantly related to the response and progression of disease at initial treatment. (6) The quality of studies is significantly related to the reliability of the conclusions, and the representativeness of the result is stronger in studies with higher quality.

Several shortcomings of this study should be acknowledged. First, all of the included studies were retrospective, and the results could be affected by uncontrolled selection and confounder biases. Second, the analysis was based on a small number of studies, and the pooled conclusions were variable. Third, this study was based on published articles, and unpublished data were not available. Therefore, publication bias is inevitable. Finally, the analysis was based on pooled data, and whether the treatment effects of re-irradiation differ based on patients’ characteristics needs to be further evaluated.

## Conclusions

In summary, this study found that re-irradiation was effective for locoregional recurrent ESCC. However, patients should be carefully monitored for grade ≥ 3 esophageal perforation, tracheoesophageal fistula, and radiation pneumonitis. Further, re-irradiation had a greater effect on survival outcomes in high-risk patients than in low-risk patients. Further prospective controlled clinical trials should be conducted to compare the efficacy and safety of re-irradiation versus non-re-irradiation for locoregional recurrent ESCC previously treated with radiotherapy.

## Supplementary Information

**Additional file 1: Fig. S1.** Sensitivity analysis for the 1-year survival rate (52.3–67.7%). CI: confidence interval; **Fig. S2.** Funnel plot for the 1-year survival rate; **Fig. S3.** Sensitivity analysis for the 2-year survival rate (20.2–27.4%); **Fig. S4.** Funnel plot for the 2-year survival rate; **Fig. S5.** Sensitivity analysis for the 3-year survival rate (15.0–28.2%); **Fig. S6.** Funnel plot for the 3-year survival rate; **Fig. S7.** Sensitivity analysis for the 5-year survival rate (6.7–18.1%); **Fig. S8.** Funnel plot for the 5-year survival rate.

## Data Availability

The datasets used and/or analysed during the current study are available from the corresponding author on reasonable request.

## Abbreviations

CIs: Confidence intervals
ESCC: Esophageal squamous cell carcinoma
