# Supplementary material for: Efficacy and safety of re-irradiation for locoregional esophageal squamous cell carcinoma recurrence after radiotherapy: a systematic review and meta-analysis
Source: Radiat Oncol. 2022 Mar 28;17:61. doi: 10.1186/s13014-022-02019-0 (PMC8962014; doi:10.1186/s13014-022-02019-0)
Supplement: Supplementary file 1 — Additional file 1: Fig. S1. Sensitivity analysis for the 1-year survival rate (52.3–67.7%). CI: confidence interval; Fig. S2. Funnel plot for the 1-year survival rate; Fig. S3. Sensitivity analysis for the 2-year survival rate (20.2–27.4%); Fig. S4. Funnel plot for the 2-year survival rate; Fig. S5. Sensitivity analysis for the 3-year survival rate (15.0–28.2%); Fig. S6. Funnel plot for the 3-year survival rate; Fig. S7. Sensitivity analysis for the 5-year survival rate (6.7–18.1%); Fig. S8. Funnel plot for the 5-year survival rate. [file 13014_2022_2019_MOESM1_ESM.docx]

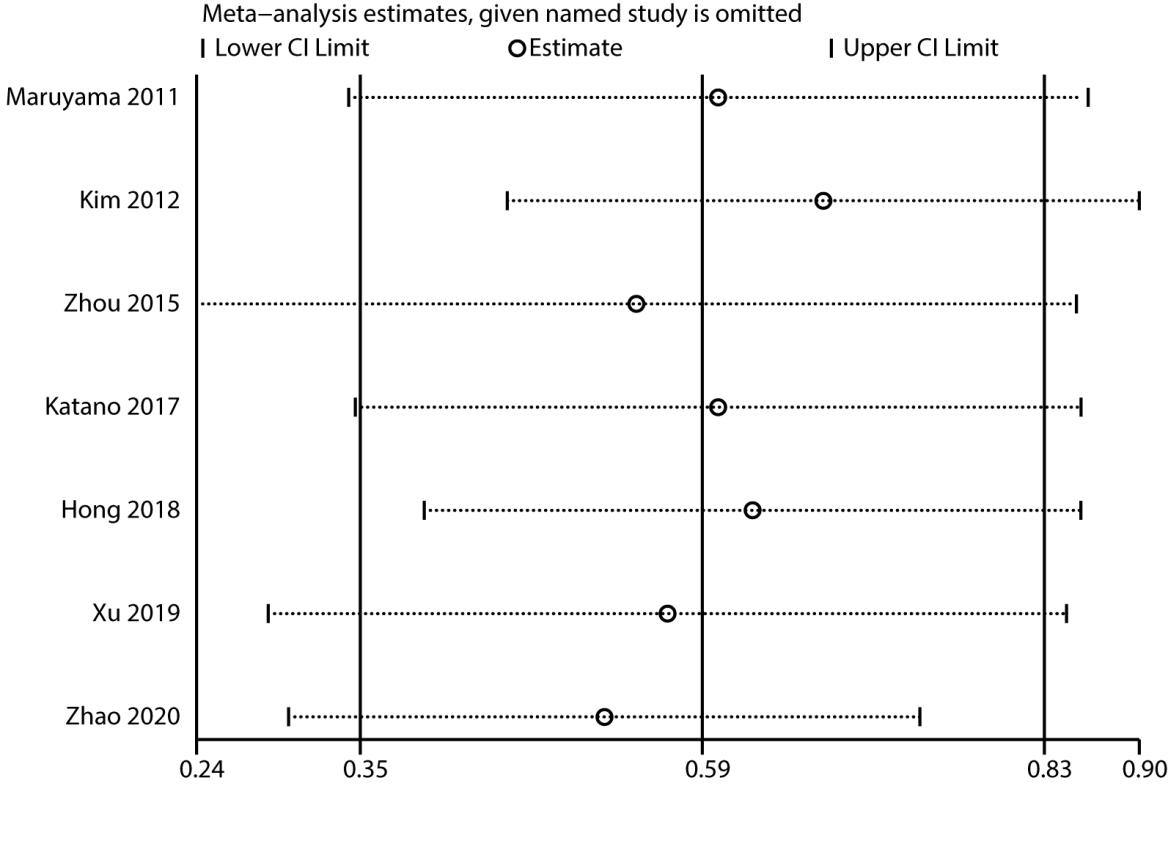


Figure S1. Sensitivity analysis for the 1-year survival rate (52.3%-67.7%)

CI: confidence interval


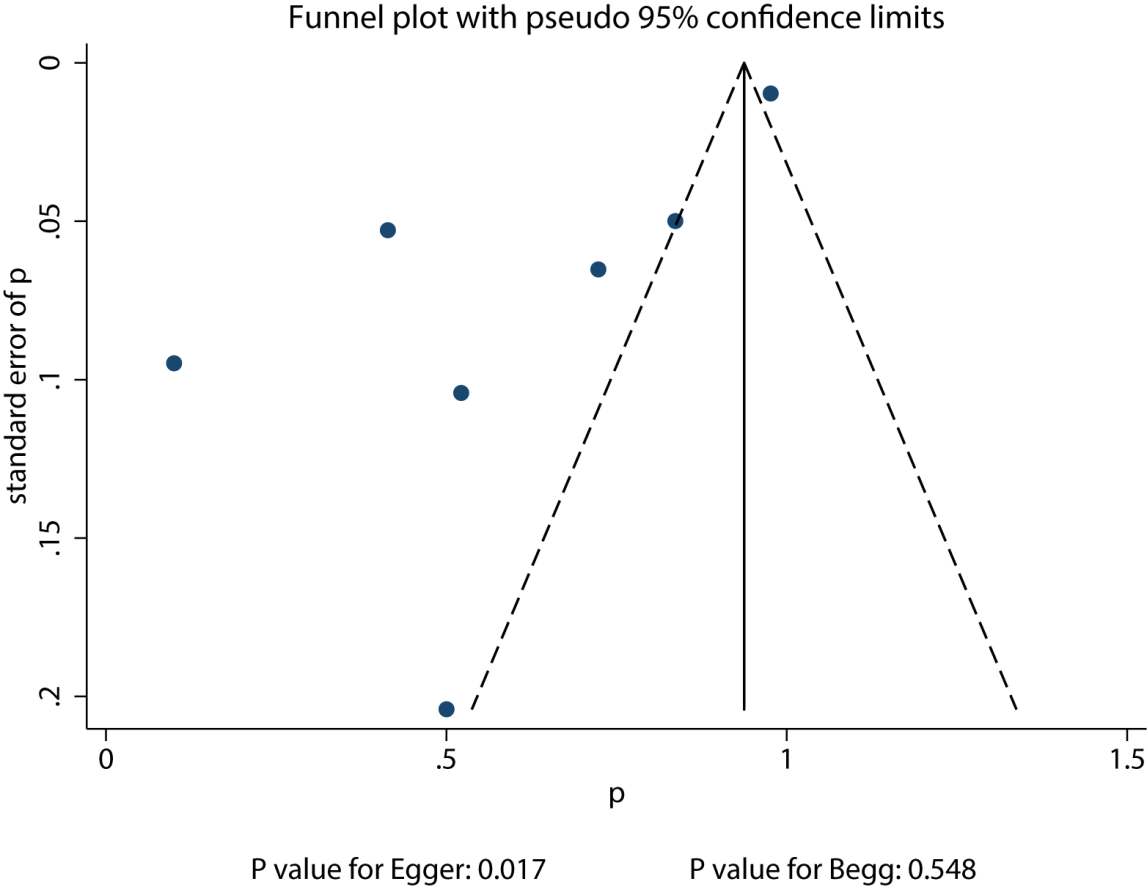


Figure S2. Funnel plot for the 1-year survival rate


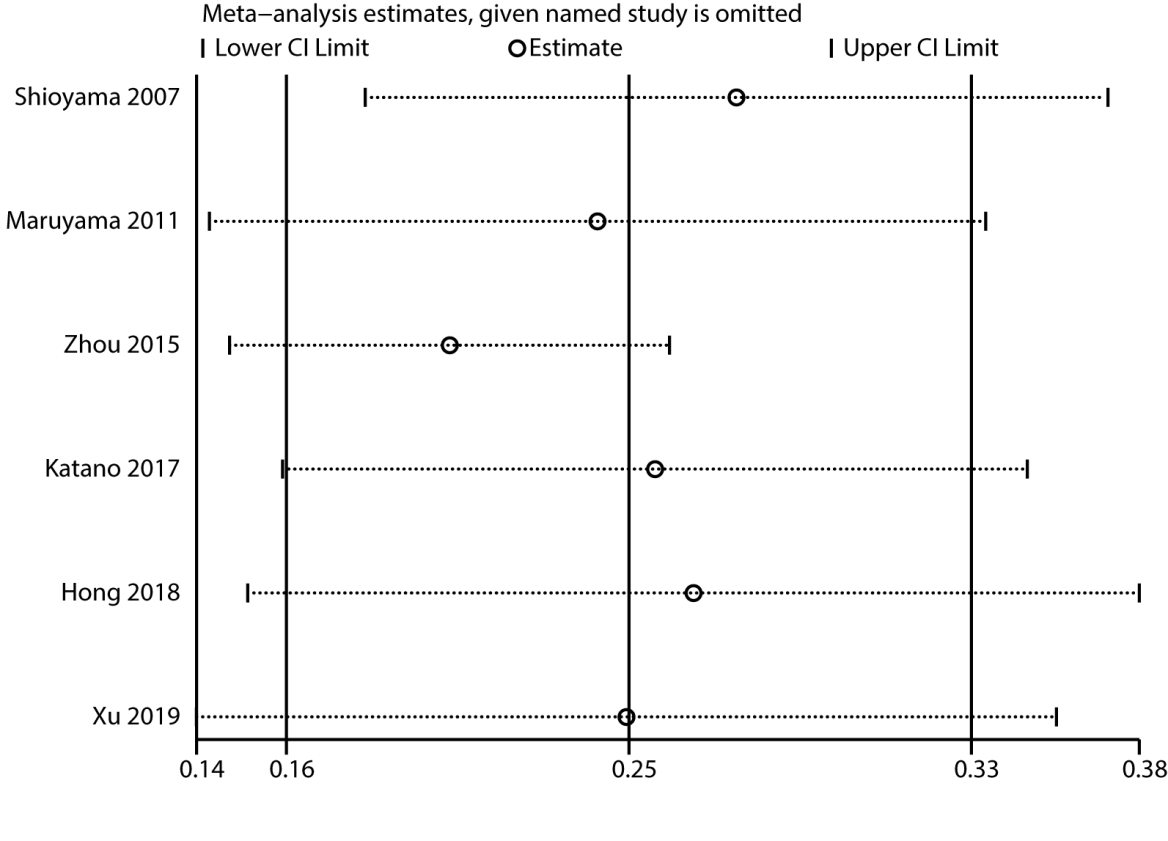


Figure S3. Sensitivity analysis for the 2-year survival rate (20.2%-27.4%)


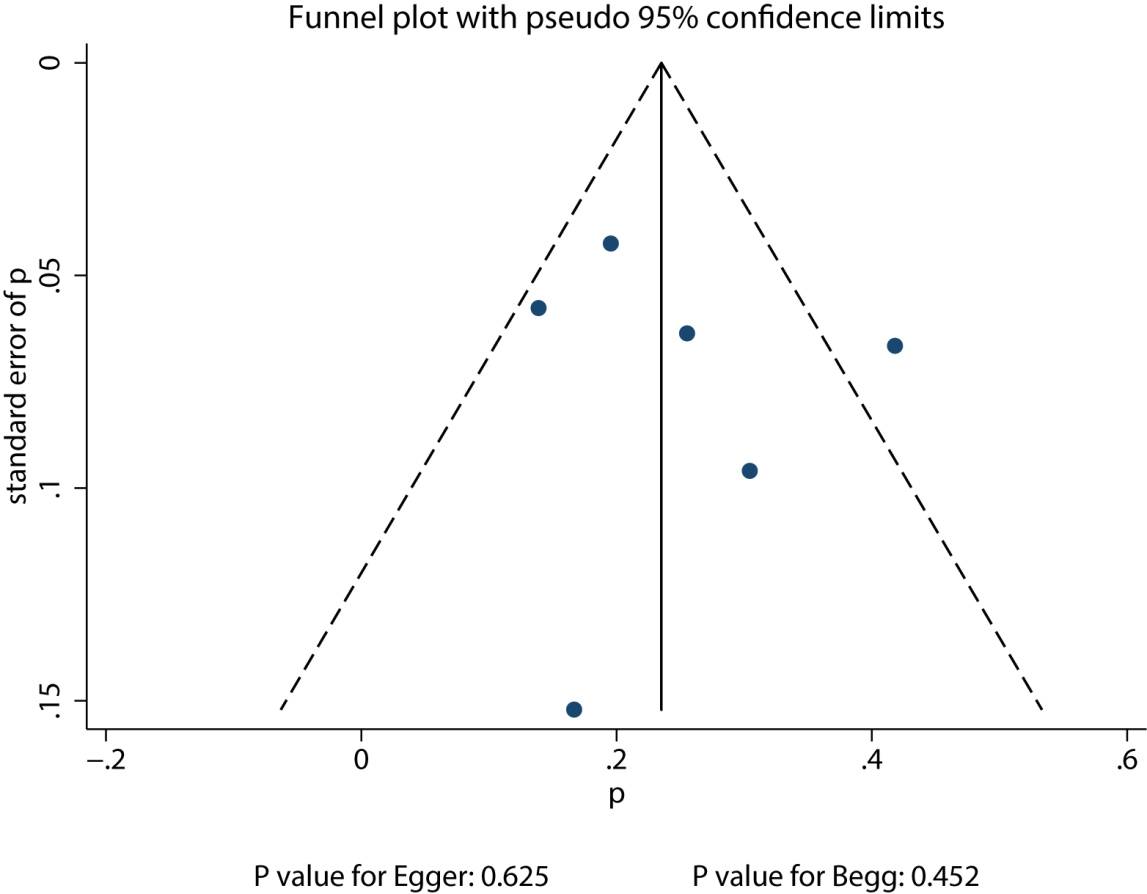


Figure S4. Funnel plot for the 2-year survival rate


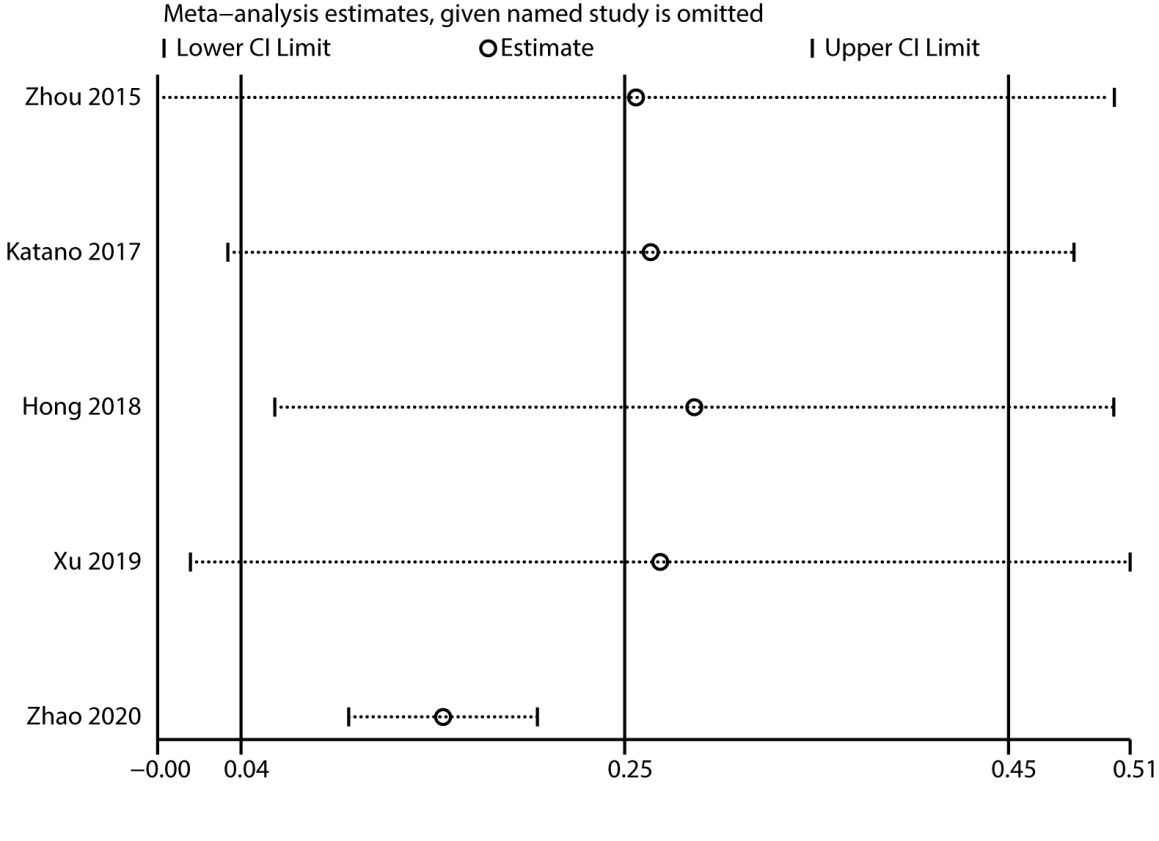


Figure S5. Sensitivity analysis for the 3-year survival rate (15.0%-28.2%)


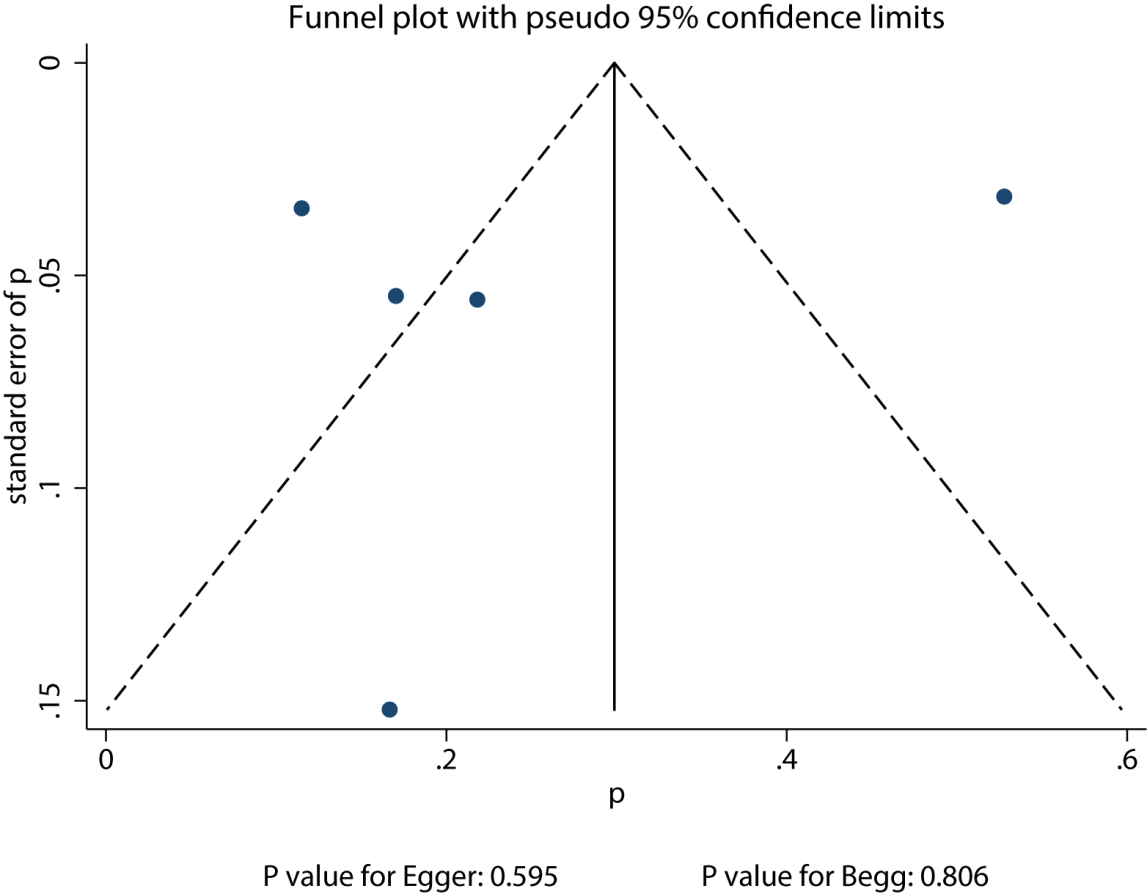


Figure S6. Funnel plot for the 3-year survival rate


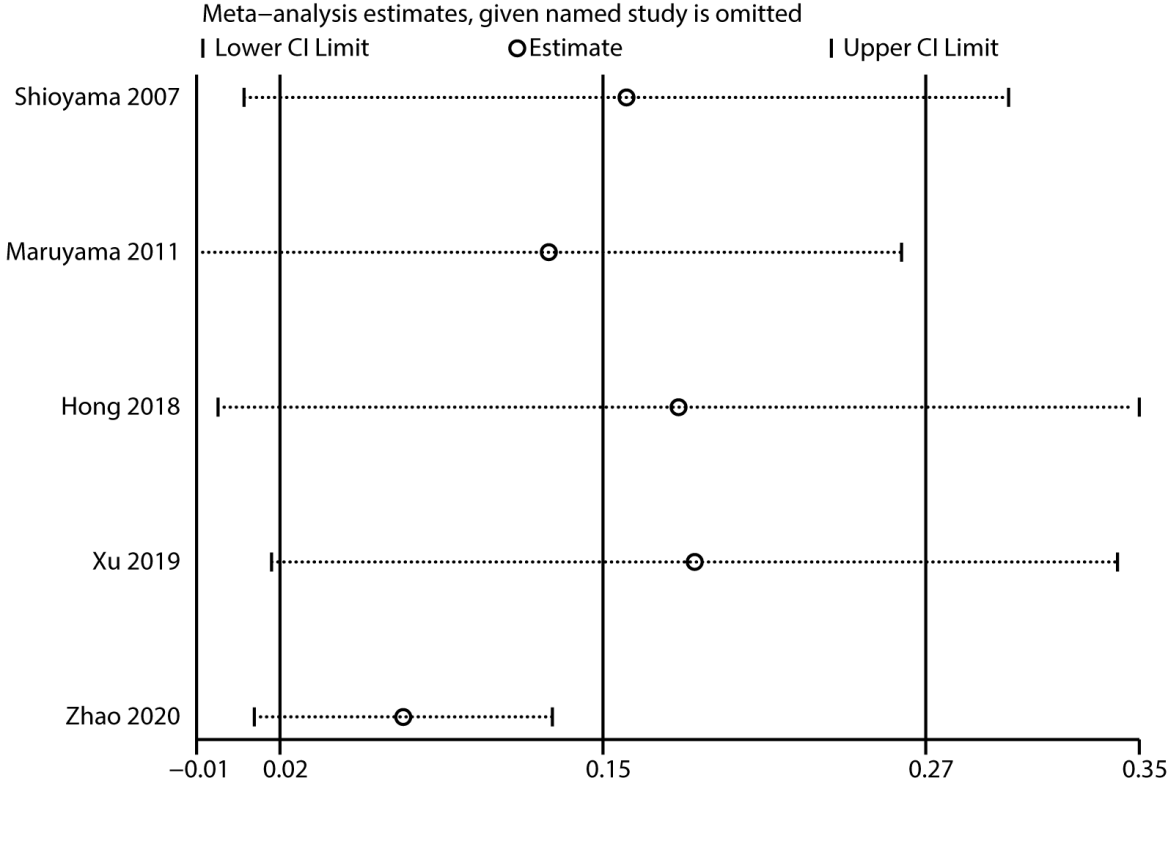


Figure S7. Sensitivity analysis for the 5-year survival rate (6.7%-18.1%)


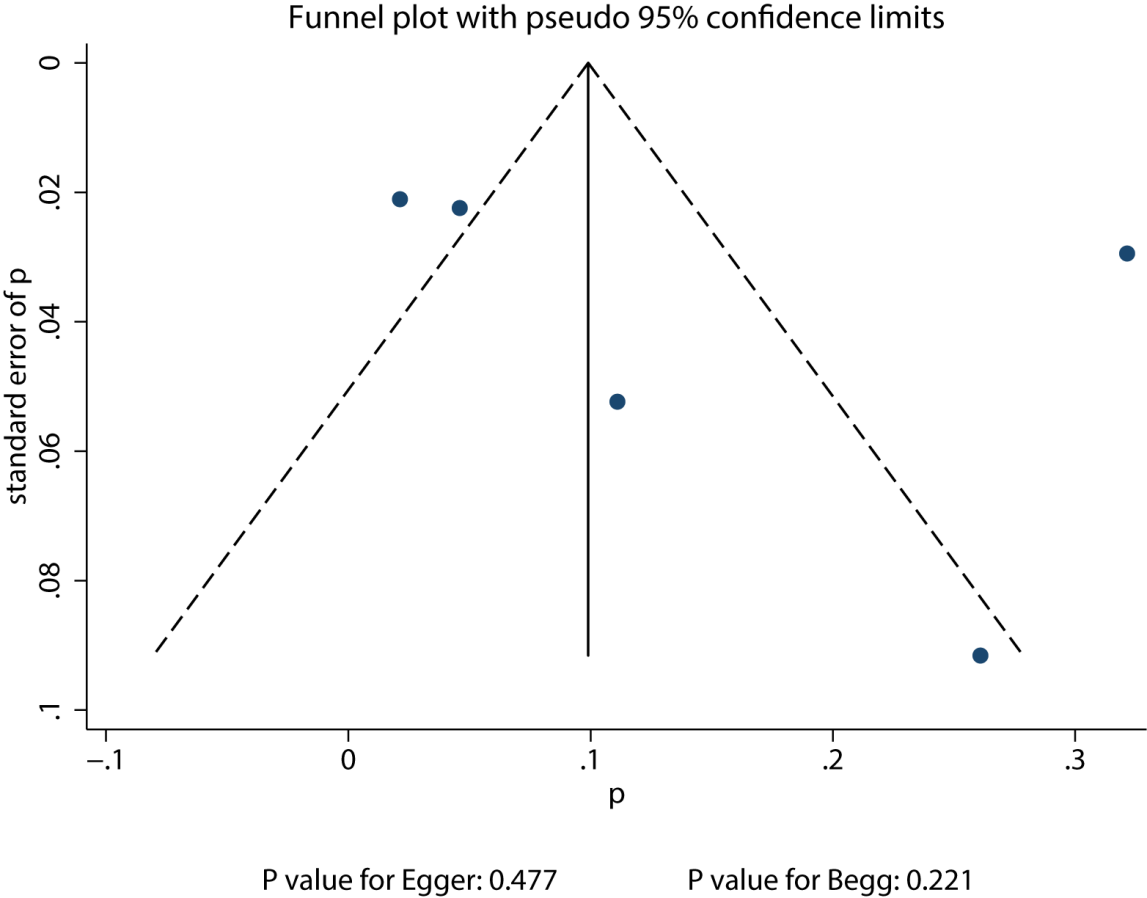


Figure S8. Funnel plot for the 5-year survival rate
